# Clinical, Hematologic, and Molecular Findings in Naturally Occurring *Babesia canis vogeli* in Egyptian Dogs

**DOI:** 10.1155/2014/270345

**Published:** 2014-02-11

**Authors:** N. Y. Salem, H. S. Farag

**Affiliations:** Department of Internal Medicine and Infectious Diseases, Faculty of Veterinary Medicine, Cairo University, Giza Square, Giza 12211, Egypt

## Abstract

*Background*. Canine babesiosis is a clinically important hemoprotozoan parasite affecting dogs. The goal of this present study was to determine the clinical symptoms and to establish its hematological and microscopic detection and compare it with the PCR findings attained from dogs infected with *Babesia canis vogeli*. *Methodology/Principal Findings*. 13-PCR confirmed Babesia-infected dogs were examined; seminested PCR was used to discover the precise type of *Babesia* and *Babesia canis vogeli* was the only subspecies detected. The most consistent clinical signs were elevated rectal temperature and a pale mucous membrane. Thrombocytopenia, monocytosis, and lymphocytosis, along with a significant reduction in red cell parameters, were the most commonly recorded hematologic alterations. Microscopic examination revealed the presence of typical large merozoites and trophozoites of *B. canis* in the ratio 76.92%. *Conclusions/Significance*. The presumptive diagnosis of canine babesiosis should be based on a fever and anemia, while thrombocytopenia is considered the hallmark of the disease; microscopic examination may not be very revealing in the detection at low parasitemia, but it remains the most rapid confirmatory method. Seminested PCR turned out to be a sensitive and accurate method for diagnosis; during the process of differentiation between *Babesia* subspecies, only *B. canis subsp. vogeli* was detected.

## 1. Introduction

Canine babesiosis is a clinically noteworthy and well-known hemoprotozoan parasite of dogs [1]. The first incidence of *Babesia* was reported in South Africa by Hutcheon in 1885 but was recognized much later in 1896 by Purvis and in 1897 by Koch [2]. *Babesia* normally causes massive injuries to the host depending on the virulence and pathogenicity of the parasite. The extent of the injuries also depends on the age and the hygiene of the host [3]. The commonly occurring *Babesia* species in dogs are *B. canis* and *B. gibsoni* [4]. *B. canis* is endemic in Southern Europe, America, Asia, and South Africa while *B. gibsoni* is found in the Middle East, Northern Africa, and South Asia [5]. In Egypt, only *B. canis* has been detected [6].

*B. canis* is a typical intraerythrocyte pear-shaped large piroplasm, frequently found in pairs within the RBCs as identified among dogs worldwide [7–9]. There are three different subspecies, *B. canis*, *B. rossi*, and *B. Vogeli*, which are morphologically alike but have different vectors and pathogenicity [10]. *R. sanguineus* is the main global vector, though *D. reticulatus* may serve as a vector for *B. canis* in Europe. Other *Ixodid* ticks belonging to the genera, namely, *Dermacentor*, *Haemaphysalis*, and *Hyalomma* are also capable of serving as vectors [11]. The life cycle of *Babesia* includes 2 stages: inside the host RBCs, in which the sporozoites convert into piroplasms, and the other inside the tick vector [10]. Clinically, the illness is characterized by fever, pale mucous membrane, anorexia, anemia, icterus, lymphadenopathy, and splenomegaly [12–15]. Clinical signs are exceedingly variable; the classical presentation is a febrile illness with perceptible anemia [16]. The severity of babesiosis varies from subclinical infection to extensive organ failure and death [1].

The most common hematologic alterations associated with *B. canis* infections are anemia and thrombocytopenia [13, 16–19].

Discrimination between subspecies of *B. canis* on the basis of blood smear examination is almost impractical. Today, polymerase chain reaction is being utilized to diagnose and distinguish between the different infections caused by various *Babesia* subspecies [20–22].

In Egypt, canines are infected by numerous infections, of which Ehrlichiosis and Babesiosis are the most significant enzootic ailments. In these areas though, dogs are pre-immunized against piroplasmosis and act as a reservoir of infection. However, this immunity may collapse under unfavorable conditions and all such animals become clinically infected [23].

The objectives of this study were to describe clinical signs and hematological alterations and to verify the results obtained by blood smear examination using molecular recognition to identify the subspecies of *Babesia* involved.

## 2. Material and Methods

Thirteen dogs of different age, sex, and breed were used in this study; the dogs were referred to a small animal medicine teaching hospital, faculty of veterinary medicine, Cairo University, Egypt, with signs attuned with babesial infection. The age, sex, breed, and locality of both control and infected dogs are listed in Table 1. The survey was conducted from March 2011 to March 2012.

The selection of control dogs depended mainly on these considerations: no prior or current tick presentation, absence of clinical signs, failure to develop the specific *Babesia* DNA amplicon on two consecutive seminested PCRs 1-month apart, and the absence of IgG in ELISA test; these animals were free from internal parasites and vaccinated.

Each dog was subjected to inclusive physical examination; the rectal temperature at the time of admission and clinical signs were recorded. During clinical testing, the presence of ticks on the coat of dogs was determined. Ticks were collected and identified [24]. Capillary blood samples collected from the ear vein and Giemsa-stained thin blood films were produced and examined under the microscope for direct detection of intraerythrocytic stages of the hemoparasite [25].

## 3. Hematologic and Molecular Study

Venous blood was collected from the cephalic vein with appropriate restrain; clinical hematology was done within 2 hours after this collection according to methods described by Feldman et al. [26]. DNA extraction was performed on the whole blood sample using Genomic DNA purification (Jena Bioscience GmbH, Jena, Germany) and DNA extraction was performed according to manufacturer's instructions. DNA extraction and PCR were performed in separate rooms.

Oligonucleotide primers (Table 2) were designed based on the canine *Babesia* 18S rRNA genes as described by Benson et al. [27]. PCR was performed according to Birkenheuer et al. [28]. The thermal cycler (Primus MWG, Bio tech, Germany) was performed for DNA amplification of the outer primer pair at the following temperatures: 95°C for 5 min, followed by 50 amplification cycles (95°C for 1 min, 56°C for 1 min, and 72°C for 1 min), and a final extension step at 72°C for 5 minutes. The seminested PCRs (i.e., specific forward primers paired with the outer reverse primer) were each carried out in separate tubes under the same conditions as the outer primer pair, except for the following: 0.5 *μ*L from the initial reaction was used as a DNA template and the reactions were amplified for 35 cycles.

A descriptive analysis using Student's *t*-test (statistica for Windows, version 5.1., StatSoft, Inc. 1984–1996) to compare between infected and control dogs was obtained; *P* value of 0.05 or lower was considered to be significant.

## 4. Results

Pertinent signalment (age, sex, and breed of the patients) are listed in Table 1. The highest infection was found in the age group of 3–5 yrs, followed by the age group of less than 3 years. German shepherd dogs were the most affected breed, and the male dogs appeared to be more infected than the female dogs.

The ticks were identified as *Rhipicephalus sanguineus* (on the basis of morphological characteristics).

The most consistent clinical signs observed during the examination were elevated rectal temperature (above 39.6°C) in 12 out of 13 dogs (92.07%), pale mucous membrane in 8/13 dogs (61.5%), anorexia 12/13 dogs (92.07%), enlargement of lymph nodes 10/13 dogs (76.92%), splenomegaly 12/13 dogs (92.07%), and presence of ticks 10/13 dogs (76.92%). Less common clinical signs included haematuria in 2/13 dogs (15.38%) and icterus in 2/13 dogs (15.38%).

**Figure 1:**
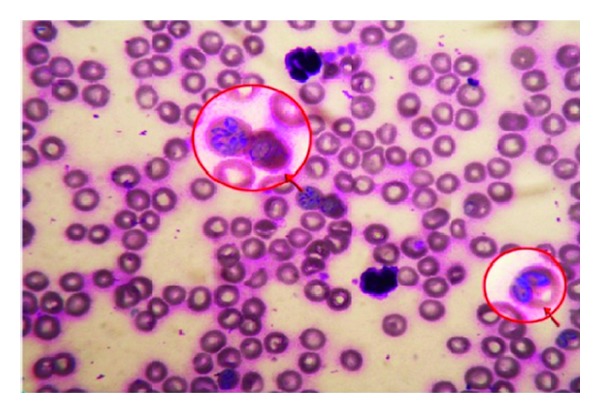
Giemsa-stained blood smear of infected dog showing the pear-shaped large *B. canis* inside the RBCs (×1000).

Examination of Giemsa-stained thin blood smear revealed the presence of typical large merozoites and trophozoites of *B. canis* (Figure 1) in 10/13 dogs (75.9%).

Hematologic findings were compared to hematologic parameters of control dogs from the same locality (Table 3). Significant reduction in RBCs, HB content, and PCV percentage (*P* < 0.05) were noted among infected dogs compared to control dogs; 61.5% of dogs showed RBCs count below reference values, while RBCs count was within reference values in 38.5% of the dogs. Haematocrite percentage was below reference values in 53.8% of dogs, while 46.2% of dogs had haematocrite within reference range. The content of hemoglobin was below normal reference values in 53.8% of dogs, while 46.2% of dogs came up with hemoglobin content within normal values.

Red cell indices showed a significant increase in MCV in 38.4% of the dogs, and 15.4% of dogs showed MCV below the normal range with 46.2% of the dogs having MCV within normal reference values. Decrease in MCHC values was noted in 38.4% of the dogs, and an increase in MCHC values in 15.4% of dogs with 46.2% of the dogs having MCHC within the normal reference values was noted.

Significant decrease in WBCs count and neutrophils along with an increase in monocyte count was noted in infected dogs compared to control dogs. Leukocytes abnormalities include neutropenia in 84.6% of dogs, lymphocytosis in 69.2% of dogs, and monocytosis in 69.2% of the dogs.

Significant decrease in platelet count was noted in infected dogs compared to control dogs. Thrombocytopenia is the most consistent hematologic abnormality observed in 100% of the affected dogs.

**Figure 2:**
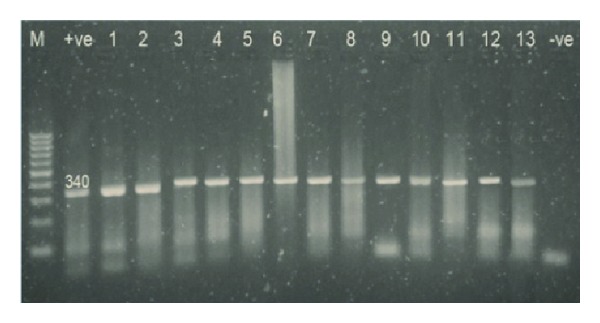
Analysis of seminested PCR products (outer primer pair only) by 1.5% agarose gel electrophoresis and ethidium bromide staining showing the positive samples after the primary amplification cycle.

During the primary reaction of seminested PCR, a 339 bp product (Figure 2) was amplified from infected canine whole blood samples (*n* = 13). During the secondary seminested reaction, the test was able to differentiate between *B. gibsoni* (Asian genotype), *B. canis subsp. vogeli*, *B. canis subsp. canis*, and *B. canis subsp. rossi*. After the second round only, *B. canis subsp. vageli* and 200 bp amplicon were visualized on the gel in all 13 samples.

## 5. Discussion

Until now, especially in Egypt, canines did not receive much attention from scientists as compared to other species, and many findings about them are still unfinished studies. Canines in this country are infected by numerous infections, of which Ehrlichiosis and Babesiosis are the most significant enzootic illnesses [23].

In this study, highest occurrence of canine babesiosis was in the age group 3–5 years followed by those below 3 years; *Babesia* infection has been known to rise with the age, reaching its peak between the age of 3 and 5 years and then peter out [30]. The male dogs reported higher infection rate than the female ones, though Martinod et al. [31] found no difference in sex susceptibility between males and females; The aggressiveness and hormonal status of male dogs may be a contributory factor here [32]. German shepherd dogs appeared to be the breed with the highest infection rate. The popularity of this breed in Egypt could be the cause for it [33].

*Rhipicephalus sanguineus* was the only tick detected in our study; *R. sanguineus* is a three-host tick and well tailored to rural areas [34] and it thrives as the biological vector of canine babesiosis [35].

The main clinical signs observed were fever, lymphadenopathy, splenomegaly, and anorexia; these signs were reported by other authors in different studies too [4, 13]. Though the clinical presentation of canine babesiosis can be highly variable, the classical presentation can be safely described as febrile illness with apparent anemia [16]. *Babesia* subspecies can cause different clinical presentations. *B. rossi* is the most virulent subspecies causing acute renal failure and acute respiratory distress syndrome [36], and *B. vogeli* is the least virulent causing mild clinical illness that may develop to severe fatal haemolytic anemia in puppies [37]. Several blood borne infections may share one or more clinical presentation(s) of *babesia*, such as *Ehrlichia spp.*, *Anaplasma spp.*, *Bartonella spp.*, *Rickettsia spp.*, and *Leishmania* [1]; thus, relying solely on the clinical signs will lead to an inaccurate diagnosis.

Febrile illness was observed in canine babesiosis-infected dogs; this may be attributed to the release of endogenous pyrogens from erythrolysis, parasitic destruction, and activation of inflammatory mediators [6]. Administration of 2 doses of imidocarb (2 weeks apart) is effective in clearing the organism from blood [14]. However, in these areas dogs are preimmunized against piroplasmosis and act as a reservoir of infection. This immunity though may collapse under unfavorable circumstances and such animals could become clinically infected [23].

When studied microscopically, a typical pear-shaped large *B. canis* was detected inside the RBCs in 10 out of the 13 blood smears; The diagnosis of canine babesiosis mainly relies on microscopic examination and a trained personnel can differentiate between *B. gibsoni* and *B. canis* based on morphology in Giemsa-stained blood films [9, 11, 38–40]. Thus, the parasite was not detected in 3 blood smears (23.07%). The divergence among the microscopic results observed may be a case of the lower sensitivity of the microscopic examination method [41]. The tick population, climatic condition, geographic region, and individual response to hemoprotozoan play important roles in the incidence of the infection [42].

Anemia, thrombocytopenia, and monocytosis were the most hematologic alterations observed. The destruction of circulating red cells by auto antibodies is directed against infected and noninfected red cell membranes resulting in intravascular and extravascular haemolysis [43–45]. However, Taboada and Lobetti [46] proposed that direct parasitic damage contributes to anemia. Nevertheless, induction of serum hemolytic factors increased erythrophagocytic activity of macrophages and damage induced by secondary immune system after the formation of antierythrocyte membrane antibodies also proved important in the pathogenesis of anemia.

Thrombocytopenia with no obvious hemorrhage observed either by the owner or during the examination, platelet destruction, increase platelet sequestration, and decrease platelet production, could be linked to thrombocytopenia [47, 48].

In Egypt, only *B. canis* was detected via PCR; *B. canis* was the common *Babesia* subspecies recorded in Egyptian dogs [6, 23, 33, 42]. *B. canis subsp. vogeli* was detected using seminested PCR, when compared to microscopic examination results; PCR proved the superiority and the sensitivity in the diagnosis and differentiation of *Babesia* infection in suspected dogs [1, 37, 49, 50]; however, the cost, equipment, and time may be a major limitation for the use of PCR in clinic-based practice.

The results of this study showed dogs with fever, lymphadenopathy, splenomegaly, anorexia, and pale mucous membrane can be suspected to carry infection related to *Babesia*. However, microscopic examination may not detect low parasitemia though; it remains the most rapid confirmatory method. Thrombocytopenia is considered the hallmark of the disease, and seminested PCR was a sensitive and accurate method for diagnosis and differentiation between *Babesia* subspecies. Only *B. canis subsp. vogeli* was detected in the course of this study.

## Figures and Tables

**Table 1:** Age, sex, breed, and locality of control and infected dogs.

Criteria	Infected dogs	Control dogs
Breed	German shepherd [10]	German shepherd [7]
	Great dane [1]	Great dane [2]
	Labrador [1]	Labrador [2]
	Malino [1]	Malino [1] Rotweiler [1]

Age	<3 yrs [4]	<3 yrs [6]
	3–5 yrs [6]	3–5 yrs [5]
	>5 yrs [3]	>5 yrs [2]

Sex	Male [10]	Male [6]
	Female [3]	Female [7]

Locality	Giza	Giza

**Table 2:** Primers sequence for canine babesiosis according to Benson et al., (2002) [27].

Primer	Sequence	Reaction and/or use	Expected amplicon
455-479F	GTCTTGTAATTGGAATGATGGTGAC	Seminested PCR outer forward primer	340 bp
793-772R	ATGCCCCCAACCGTTCCTATTA	Seminested PCR outer reverse primer	
BgibAsia-F	ACTCGGCTACTTGCCTTGTC	Seminested PCR *B. gibsoni* (Asian genotype) specific forward primer	185 bp
BCV-F	GTTCGAGTTTGCCATTCGTT	Seminested PCR *B. c. vogeli *specific forward primer	192 bp
BCC-F	TGCGTTGACGGTTTGACC	Seminested PCR *B. c. canis *specific forward primer	198 bp
BCR-F	GCTTGGCGGTTTGTTGC	Seminested PCR *B. c. rossi *specific forward primer	197 bp

**Table 3:** Hematologic findings of 13 dogs with babesiosis from small animal internal medicine teaching hospital, faculty of veterinary medicine, Cairo University.

Parameter	Patient data	Control data^1^	Reference range^2^
RBCs (×10^6^/*μ*L)	4.05 ± 0.8^a^	6.41 ± 1.09^b^	5.5–8.5
Hemoglobin (g/dL)	11.375 ± 0.61^a^	14.74 ± 2.07^b^	12.0–18.0
PCV (%)	32.33 ± 7.00^a^	43.14 ± 6.44^b^	37–55
MCV (fL)	74.67 ± 13.2	68.14 ± 6.86	66–77
MCH (pg)	25.25 ± 5.46	23.01 ± 2.92	21.0–26.2
MCHC (%)	32.17 ± 3.72	33.56 ± 1.77	32.0–36.3
WBCs (×10^3^/*μ*L)	9.650 ± 1.4^a^	12.850 ± 1.61^b^	6,000–17,000
Neutrophil (×10^3^/*μ*L)	3.787 ± 0.92^a^	6.728 ± 0.89^b^	3,000–11,500
Lymphocyte (×10^3^/*μ*L)	4.583 ± 1.33	4.928 ± 0.37	1,000–4,800
Monocyte (×10^3^/*μ*L)	1.062 ± 0.196^a^	0.771 ± 0.13^b^	0.1–1.4
Platelets (×10^3^/*μ*L)	93.667 ± 15.0^a^	268.93 ± 14.2^b^	200,000–500,000

^(ab)^Different letters on the same line indicate statistically different means (*P* < 0.05); confidence interval: 95%.

^1^Control data was done from apparently healthy dogs tested negative on two PCR cycles with 1-month period; ^2^Rizzi et al. [29].
